# A Genetic Tool to Identify Predators Responsible for Livestock Attacks in South America and Recommendations for Human–Wildlife Conflict Mitigation

**DOI:** 10.3390/ani14060838

**Published:** 2024-03-08

**Authors:** Eduardo A. Díaz, María José Pozo, Pablo Alarcón, Gabriela Pozo, Rebecca Zug, Carolina Sáenz, Maria de Lourdes Torres

**Affiliations:** 1 Escuela de Medicina Veterinaria, Colegio de Ciencias de la Salud, Universidad San Francisco de Quito (USFQ), Calla Diego de Robles y Avenida Pampite, Cumbayá, Quito 170901, Ecuador; eadiaz@usfq.edu.ec; 2 Laboratorio de Biotecnología Vegetal, Colegio de Ciencias Biológicas y Ambientales, Universidad San Francisco de Quito (USFQ), Calle Diego de Robles y Avenida Pampite, Cumbayá, Quito 170901, Ecuador; mpozo@usfq.edu.ec (M.J.P.); palarconb@usfq.edu.ec (P.A.); gpozo@usfq.edu.ec (G.P.); 3 Laboratorio de Carnívoros, Colegio de Ciencias Biológicas y Ambientales, Universidad San Francisco de Quito (USFQ), Quito 170901, Ecuador; rlzug@usfq.edu.ec; 4 Escuela de Medicina Veterinaria, Instituto de Biodiversidad Tropical IBIOTROP, Hospital de Fauna Silvestre Tueri, Universidad San Francisco de Quito USFQ, Quito 170901, Ecuador; csaenz@usfq.edu.ec

**Keywords:** domestic dog, human–large carnivore coexistence, molecular identification, retaliatory killing, saliva samples

## Abstract

**Simple Summary:**

Livestock predation fuels conflicts between humans and wildlife, leading to the killing of wild animals such as Andean bears, jaguars, and pumas. Despite wild predators being blamed, domestic dogs also harm livestock and spread diseases among animals and people, affecting nature, local livelihoods, food, and our well-being, the very goals the United Nations aims to safeguard by 2030. In Ecuador, where families depend on livestock, retaliatory hunting jeopardizes the survival of wildlife. However, the role of dogs in these conflicts remains unclear. This study analyzed DNA found on bite wounds, revealing traces of dog saliva on animals presumed to be attacked by wild predators. This discovery challenges misconceptions about these animals in Ecuador and emphasizes the need to manage dog populations more effectively. To address this issue, we propose incorporating DNA tests in livestock predation cases to assess the involvement of dogs accurately. By understanding the true causes, strategies can be devised to mitigate these conflicts, preserving the vital role of these important animals in our ecosystem.

**Abstract:**

Livestock predation induces global human–wildlife conflict, triggering the retaliatory killing of large carnivores. Although domestic dogs (*Canis familiaris*) contribute to livestock depredation, blame primarily falls on wild predators. Dogs can also transmit pathogens between wildlife, domestic animals, and humans. Therefore, the presence of free-ranging dogs can have negative consequences for biodiversity conservation, smallholder economy, food supply, and public health, four of the United Nations’ Sustainable Developed Goals (SDGs) for 2030. In Ecuador, where livestock sustains rural households, retaliatory poaching threatens Andean bear (*Tremarctos ornatus*), jaguar (*Panthera onca*), and puma (*Puma concolor*) populations. However, the role of dogs in these incidents remains underexplored. The present study evaluates the possibility of reliable molecular identification of predatory species from DNA traces in bite wounds. Our results revealed the presence of dog saliva on four out of six livestock carcasses presumably attacked by wild predators. These findings highlight the importance of rectifying misinformation about large carnivores in Ecuador and the need to control dog populations. We recommend that local administrations incorporate DNA analysis into livestock predation events to examine how common the problem is, and to use the analysis to develop conflict mitigation strategies which are essential for the conservation of large carnivores.

## 1. Introduction

Domestic dogs (*Canis familiaris*) occur in every country in the world and on every continent except Antarctica [1], where they were removed because of the threats they posed to native wildlife [2]. Dogs range from owned and completely dependent on humans to feral and free-ranging, with an estimated global population of 1 billion and 100 million, respectively [3]. The process of domestication began several thousand years before that of any other mammal, and dogs have accompanied humans for more than 15,000 years [4]. Hundreds of studies have shown that free-ranging dogs have become a problem for both wildlife conservation and public health. From a conservation perspective, their impacts include predation, disturbance, disease transmission, competition, and hybridization [1,5]. In terms of human health, dogs are sometimes responsible for road traffic accidents, bite injuries, and a source of zoonotic pathogens [6,7,8]. In recent years, there has been growing concern about the socio-economic impact of free-ranging dog attacks on livestock, as they may be the main cause of animal loss for small farms, posing a threat to the livelihoods of vulnerable groups [9,10,11]. In addition, small-scale livestock production plays a fundamental role as a source of income and nutrition for households in developing countries like Ecuador [12,13]. Therefore, the presence of uncontrolled free-ranging dogs can have a negative impact on biodiversity conservation, public health, the economy, and food supply, 4 of the 17 Sustainable Development Goals (SDGs) created by the United Nations “to achieve a better and more sustainable future for all” [14].

The control of dog populations can be particularly challenging due to their close relationship with humans and the perception that they are domesticated. However, domestic dogs can revert to a feral state with far greater negative impacts than previously thought [5,15,16]. For example, studies show that when dogs coexist with wild carnivores, they are responsible for attacks wrongly attributed to wildlife, resulting in negative public opinion, which could encourage persecution and the illegal killing of protected wild carnivores [17,18,19]. Some studies have attempted to distinguish between potential predators by using attack and consumption patterns [20,21] or tooth marks [22,23]. However, there is often an overlap between the attack characteristics of different predator species and variability in the experience and response time of field technicians, making species discrimination prone to observer bias and uncertainty [24,25]. Identification from the visual examination of livestock wounds can be ambiguous, and genetic analysis is highly preferred for the accurate identification of predatory species [10,26]. The genetic identification of predators from saliva collected around prey bite wounds has been used successfully to identify leopards (*Panthera pardus*) and tigers (*Panthera tigris*) in Asia [27,28], lynxes (*Lynx lynx*) and wolves (Canis lupus) in Europe [10,18,26,29], and black bears (*Ursus americanus*), bobcats *(Lynx rufus*), and coyotes (*Canis latrans*) in North America [24,30,31,32]. Determining the species involved in an attack is important because conflict mitigation solutions vary between species. For example, electric fencing around pastures has been successful for preventing some bear species from accessing livestock [33], but these low fences would not work for pumas who can jump more than 5 m vertically. Other methods, such as night corrals, and the specific management of newborn and young individuals would be more effective for this species [34]. Nevertheless, to date, we have not found any study that has used saliva samples for the molecular identification of predators in South America, even though attacks on livestock are common throughout the continent [35,36,37,38,39,40,41].

Ecuador is one of the megadiverse countries of the world, but it is also one of the countries with the highest number of threatened species on the planet [42]. The three large carnivores in South America, Andean bear (*Tremarctos ornatus*), jaguar (*Panthera onca*), and puma (*Puma concolor*), are present in this country, and they all are listed as endangered (EN) on the Red List of Mammals of Ecuador [43]. These large carnivores are often considered predators of domestic animals in ranching areas, and pre-emptive killings or retaliation for attacks on livestock are one of the main causes of their population decline [44,45,46]. Recent field observations using transects and camera traps in the Andean region of Ecuador have shown that the presence of feral dogs alters activity patterns and reduces the abundance of native mammals [47,48]. There is also data from dog attacks on wild species [49,50,51], but, to date, there are no published studies that have investigated whether dogs may also be negatively interacting with livestock. The Ministerio de Ambiente, Agua y Transición Ecológica (MAATE) is making efforts to manage human–wildlife conflicts through the training of its agents and the publication of a guide that explains how to determine if an attack on livestock was from an Andean bear, a jaguar, a puma, or a domestic dog [52]. Nevertheless, the conservation of large carnivores requires molecular techniques that allow for a reliable identification since field observations often underestimate the role of dogs in attacks [10].

The use of saliva genotyping has widely demonstrated the relevant role of domestic dogs in attacks on livestock [9,10,11,17,18,25], but to the best of our knowledge, there are no related data in Ecuador. Therefore, given the lack of a useful tool for the reliable identification of predators in the Andean region, and that preventive killings and retaliation for attacks on livestock are one of the main causes of their population decline, we explored for the first time the possibility of recovering saliva from livestock bite wounds to genetically identify Andean bears, jaguars, pumas, and domestic dogs. The objective was to obtain a reliable, easy-to-use tool that aids in the identification of livestock predators, prevents the exacerbation of human–large carnivore conflict in the region, and raises awareness of the role of domestic dogs in these attacks. Furthermore, since mitigating conflicts associated with livestock predation are essential to conserving large carnivores in human-dominated landscapes, we recommend strategies to prevent livestock predation as a contribution to achieving the SDGs.

## 2. Materials and Methods

To collect genetic samples from Andean bears, pumas, and jaguars, we worked closely with captive centers in Ecuador. Non-invasive saliva samples were collected from animals at the Zoológico de Quito (ZQ), Eco Zoológico San Martín (EZSM), and the Zoo Bioparque Amaru (ZBA) during enrichment activities. All samples were collected and transported with permission from the MAATE (Contrato Marco No. MAE-DNB-CM-2019-0118). Samples were processed at the Laboratorio de Biotecnología Vegetal of Universidad San Francisco de Quito (LBV-USFQ), where they were analyzed, and protocols were developed.

Our study consisted of three phases: 1. DNA extraction and PCR protocol standardization for the species of interest, 2. Simulated attacks in captive centers to test the standardized protocols, and 3. Field testing of this protocol to confirm its effectiveness in a real-world setting.

### 2.1. Phase One: PCR Protocol Standardization

To standardize PCR protocols, species-specific primers were employed for each carnivore species: Andean bear, domestic dog, jaguar, and puma. Saliva samples were obtained from Andean bears, jaguars, and pumas in captive centers (ZQ, EZSM, ZBA). Samples of domestic dogs were obtained from pets. Saliva samples were collected by swabbing the animal’s gums during enrichment activities. The swabs were then placed in microtubes containing DNA stabilization buffer [53] and immediately transported to the LBV-USFQ, where DNA was extracted using the PureLink Genomic DNA Mini Kit (Thermo Fisher Scientific, Waltham, MA, USA). The extraction process followed the manufacturer’s instructions with one modification: we increased the incubation time in the lysis step from 10 to 60 min. Cow DNA from supermarket meat was also extracted as a control. DNA concentration and quality were assessed using a Nanodrop 2000 Spectrophotometer and visualized in a 1% agarose gel. Species-specific primers found in the literature (Table 1) were synthesized for all species (Andean bear, jaguar, puma, domestic dog, and cow), and PCRs were run using DNA from each species to standardize protocols and verify that there was no cross-species amplification. The standardized PCR protocols for each carnivore species were also tested on livestock that could potentially be attacked: chickens, donkeys, llamas, pigs, and sheep.

### 2.2. Phase Two: Testing the Standardized Protocols in Simulated Attacks

Andean bears, jaguars, and pumas in captivity at EZSM, along with domestic dogs, were provided with pieces of cow meat to chew on. Saliva samples were swabbed from the chewed meat, placed in microtubes containing DNA stabilization buffer, and transported to LBV-USFQ. DNA was then extracted using the PureLink Genomic DNA Mini Kit (Thermo Fisher Scientific, Waltham, MA, USA). All standardized PCR protocols were tested on each sample in a blind assay to determine if the species could be correctly identified. This assay was repeated five times. 

### 2.3. Phase Three: Use of Standardized Protocols to Analyze DNA from Bite Wounds Resulting from Attacks on Livestock in Rural Areas of Ecuador (Field Testing)

This phase constitutes an ongoing collaboration with field experts from various agencies, such as the Unidad Nacional de Policía de Protección del Ambiente (UPMA) and MAATE. We are conducting training sessions on how to correctly collect, store, and transport samples after an attack. Upon receiving a report of a livestock attack, a person (preferably previously trained) visits the attack site, takes swabs from the wounds of the animal, and places them in microtubes containing DNA stabilization buffer. These samples are then transported to LBV-USFQ. DNA is extracted using the PureLink Genomic DNA Mini Kit (Thermo Fisher Scientific, Waltham, MA, USA) and the standardized protocols outlined above are run to identify whether DNA from a species of carnivore or dog is present in the bite wounds from the attacked animal. Subsequently, the laboratory provides a written report with the results.

## 3. Results

### 3.1. Phase One: PCR Protocol Standardization

For each species, we obtained at least one species-specific primer pair and developed a standardized PCR protocol (Table 1). These protocols allowed for the successful amplification of the targeted DNA region from saliva samples. The PCR protocol for cow DNA amplification, developed by [58], was successfully used as a control when amplifying DNA from tissue samples (supermarket cow meat). As livestock other than cows are sometimes attacked, all PCR protocols were also standardized for commonly found livestock such as chickens, donkeys, llamas, pigs, and sheep to verify that there was no cross-species amplification.

### 3.2. Phase Two: Testing the Standardized Protocols in Simulated Attacks

**Figure 1:**
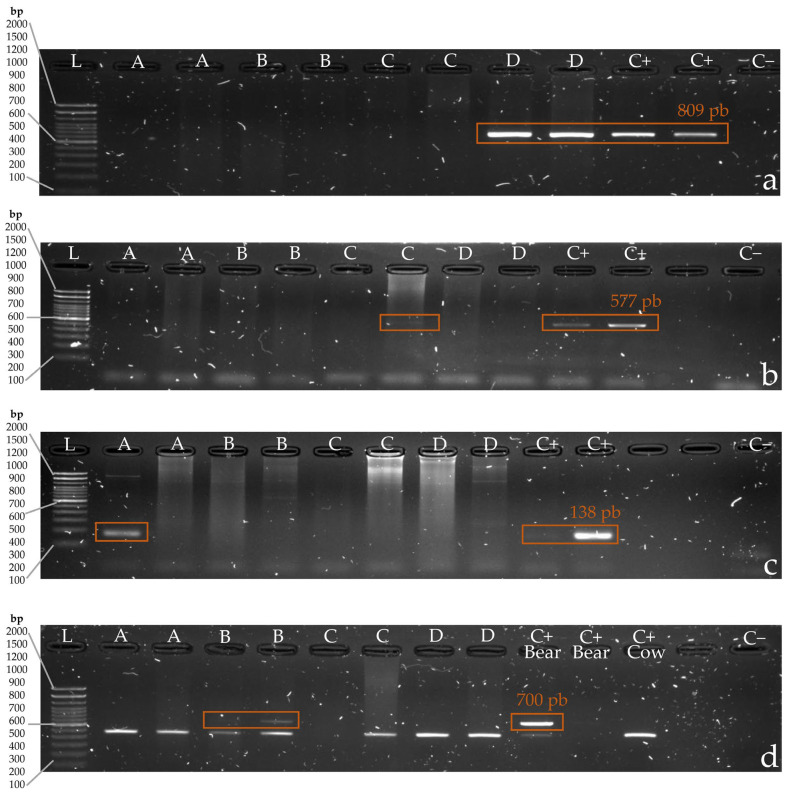
Agarose electrophoresis gel results of blind assay 3. For each gel, L corresponds to DNA ladder, C+ corresponds to positive control and C− corresponds to negative control and A, B, C, and D correspond to samples from saliva from an unknown species from blind assay 3 (jaguar, puma, Andean bear, or dog). (**a**) Amplification of expected band size of approximately 809 base pairs using dog-specific primers (CytB). (**b**) Amplification of expected band size of approximately 577 base pairs using jaguar-specific primers (12S). (**c**) Amplification of expected band size of approximately 138 base pairs using puma-specific primers (16S). (**d**) Amplification of expected band size of approximately 700 base pairs using Andean bear-specific primers (COI).

The developed PCR protocols were effective in the detection of most simulated attacks, and blind assay number 3 successfully identified DNA from all four carnivores (Table 2). In blind assay 1, DNA was identified from three of the four carnivore species (excluding jaguar), and in assays 2 and 5, DNA was identified from two carnivore species (Andean bear, domestic dog). In blind assay 4, DNA was identified from only the domestic dog. The gel electrophoresis results from blind assay 3 can be seen in Figure 1.

### 3.3. Phase Three: Use of Standardized Protocols to Analyze DNA from Bite Wounds Resulting from Attacks on Livestock in Rural Areas of Ecuador (Field Testing)

To date, we have analyzed samples from six livestock attacks. The first attack took place in Nono, Quito, Pichincha (0°06′54″ S 78°32′21″ W). A calf was attacked and killed at an unknown date, and samples were taken from wounds in the neck and back extremities on 27 December 2021 using the methods described above and transported to LBV-USFQ. DNA was extracted and amplified in four separate reactions using the species-specific primers and standardized protocols. All reactions included negative and positive controls to corroborate the results. We were able to detect domestic dog DNA and puma DNA in the samples taken from neck wounds (Table 3).

**Figure 2:**
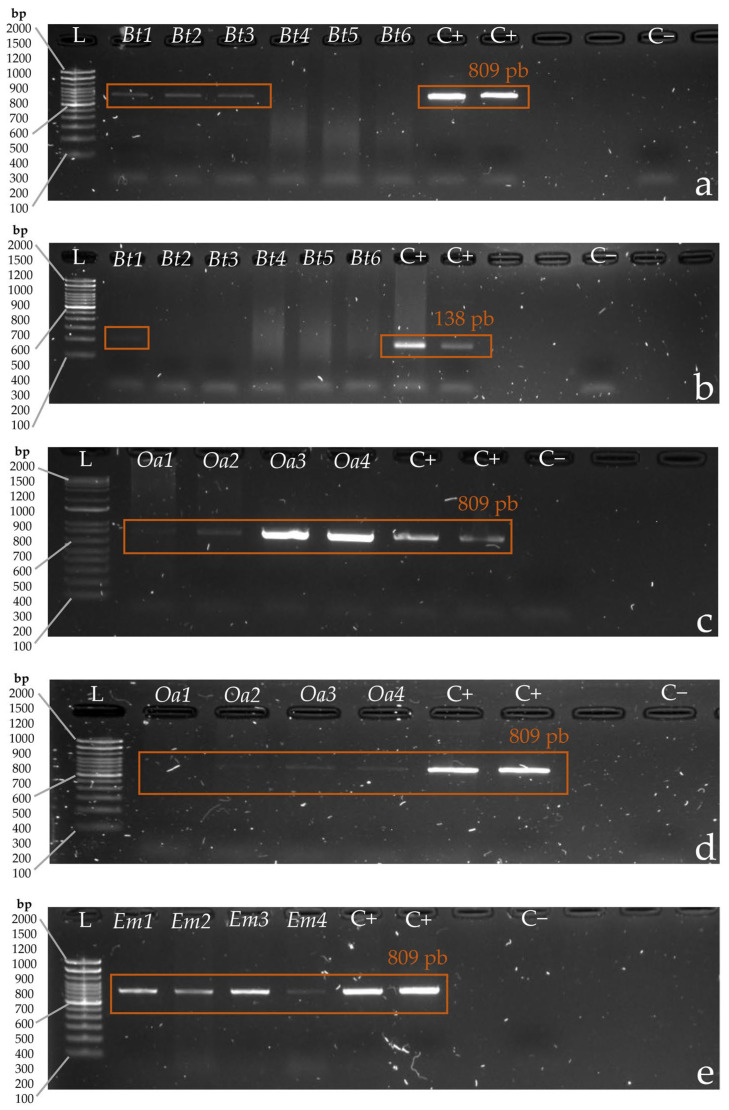
Electrophoresis gels of DNA amplifications in samples from Attack 1 and 2. For each gel, L corresponds to DNA ladder, C+ corresponds to positive control and C− corresponds to negative control. (**a**) Attack 1. Identification of dog DNA (*Canis familiaris*), where *Bt1*, *Bt2*, *Bt3*, *Bt4*, *Bt5*, and *Bt6* correspond to samples taken from the wounds of the attacked calf. Expected band size: ~809 bp. (**b**) Attack 1. Identification of puma DNA (*Puma concolor*), where *Bt1*, *Bt2*, and *Bt3* correspond to samples taken from the wounds of the attacked calf (*Bos taurus*). Expected band size: ~138 bp. (**c**) Attack 2. Identification of dog DNA (*Canis familiaris*), where *Oa1*, *Oa2*, *Oa3*, and *Oa4* correspond to samples taken from the wounds of each of the attacked sheep (*Ovis aries*). Expected band size: ~809 bp. (**d**) Attack 5. Identification of dog DNA (*Canis familiaris*), where *Oa1*, *Oa2*, *Oa3*, and *Oa4* correspond to samples taken from a single wound on the neck of the attacked sheep (*Ovis aries*). Expected band size: ~809 bp. (**e**) Attack 6. Identification of dog DNA (*Canis familiaris*), where *Em1*, *Em2*, *Em3*, and *Em4* correspond to samples taken from wounds of the attacked mule (*Equus mulus*). Expected band size: ~809 bp.

The second attack occurred in Yanahurco Grande, Saquisilí, Cotopaxi (0°52′56″ S 78°45′37″ W), where four sheep were attacked and killed on 20 February 2022. Samples were taken from wounds on the four individuals by swabbing and were transported to LBV-USFQ on 21 February 2021. DNA was extracted, and amplifications were performed following the same protocol described for Attack 1. Electrophoresis gels of DNA amplifications from samples from Attacks 1 and 2 can be seen in Figure 2. We were able to detect domestic dog DNA in all four samples (Table 3).

Attacks 3 and 4 took place in Paluguillo, Pichincha (0°16′24″ S 78°15′12″ W) and Nono, Pichincha (0°03′29″ S 78°34′12″ W), respectively. In Attack 3, one sheep was killed on or before 25 December 2022, and samples were taken from a single wound on its face on 27 December 2022 using the standardized methods. In Attack 4, one cow was attacked and killed before or on 23 April 2023, and samples were taken on 26 April 2023 from its face, thorax, and leg using the standardized methods. DNA was extracted from both attacks and amplified using the species-specific primers and standardized protocols. No carnivore DNA was found in either attack (Table 3).

Attack 5 took place in Chilla, El Oro (3°27′54.3″ S 79°34′35.6″ W), where one sheep was killed on 22 December 2023. Samples were taken from a single bite wound from the sheep’s neck on 22 December 2023 using standardized methods. DNA was extracted, and amplifications were performed following the same protocol described for Attack 1. We were able to detect domestic dog DNA in the provided samples (Figure 2d).

Attack 6 took place in Las Villegas, Santo Domingo (0°03′04.3″ S 79°28′15.2″ W). One mule was attacked and killed on 14 January 2024. Samples were taken by an untrained person who did not follow the standardized protocol. Twenty-three swabs were collected from wounds on the mule’s mouth, nose, head, and hip on 14 January 2024 and were stored in two plastic cups due to the unavailability of microtubes with DNA stabilization buffer. Additionally, the bite wound from which each swab was taken was not identified. DNA was extracted from all swabs, and amplifications were preformed following the protocol described for Attack 1. Dog DNA was detected in all samples (Figure 2e).

## 4. Discussion

Previous studies show that domestic dogs are responsible for wildlife predation in Ecuador [49,50], and that attacks to wildlife are becoming more frequent, probably due to the increase in free-ranging dog densities [51]. However, until now, no data have been published on their involvement in livestock predation. This information gap is relevant, as false perceptions about large carnivores in the region may be increasing negative public attitudes and triggering preventive and retaliatory poaching [34,59,60]. As mentioned previously, conflicts over livestock predation are one of the main drivers of the decline in Andean bear, jaguar, and puma populations in Ecuador [44,45,46]. We evaluated the possibility of identifying predator species from salivary DNA remains in livestock bite wounds. Our results show that the saliva sampled from the wounds of four out of the six attacks involving one calf, several sheep, and one mule, presumably attacked by wild predators, contained DNA from domestic dogs (Table 3). Additionally, in the case of calf predation, puma DNA was also detected. Thus, this research demonstrates the feasibility of identifying predator species DNA from bite wounds and highlights that it cannot be assumed that Andean bears, jaguars, or pumas are always responsible for attacks on livestock in the region.

Molecular identifications of salivary DNA are increasingly applied in wildlife forensic investigations, rapidly improving the understanding of predator–prey interactions. The reviewed literature shows that salivary DNA genotyping has often been used to discriminate predation on livestock by domestic dogs versus wildlife [9,10,11,17,18,25], but it is the first time that molecular identification from non-invasive saliva samples has been successfully used in Ecuador. Until now, there was a field guide to visually discriminate attacks on livestock by bears, dogs, jaguars, and pumas in Ecuador [52]. Identification from the visual examination of livestock wounds can be ambiguous, as overlap between attack characteristics of different predators makes species discrimination prone to observer bias, especially due to delayed response time, insufficient experience, or if a carcass has been visited by several predator and/or scavenger species [24]. Genetic analysis, supported by detailed field reports and necropsies, is highly preferred for accurate identification [10,26,61]. In the present study, we identified DNA saliva from dog and puma in the calf carcass, but we could not deduce the identity of the initial predator versus a scavenger. We were also unable to recover high-quality predator DNA from two attack events. Therefore, all stakeholders in attack investigations must be trained to identify signs left by predators, attack patterns, and consumption of carcasses, as well as apply standardized sampling and necropsy protocols to achieve reliable predator identification. For example, reducing the time between a predation event and sample collection prevents DNA degradation and improves the detection rate, as demonstrated in a study conducted by [25] and corroborated by our results. We successfully detected DNA from carnivore species in Attacks 1, 2, 5, and 6, where the time lapse between the predation event and sample collection was one day or less. However, we were unable to detect carnivore DNA in Attacks 3 and 4, where the time lapse between the predation event and sample collection was at least two days in Attack 3 and at least three days in Attack 4 (Table 3). DNA contaminations from multiple predators might be avoided by collecting swab samples from wounds caused by a single tooth [18], obtaining multiple swabs from each puncture can ensure that enough DNA is recovered for genetic analysis [31], and collecting hemorrhaged bites is the best way to obtain DNA from the predator and not a scavenger [61].

Predation by Andean bears, jaguars, and pumas is a long-standing problem throughout their range, but it is not random; conflict escalates when grazing areas overlap with predator territory and livestock protection measures and management are inadequate for the area [62,63]. Ecuador suffers the highest deforestation rate in South America, especially in the buffer zones around protected areas, where pastures are one of the most important drivers of deforestation [64]. The demand for meat and milk from a growing population has caused a rapid and uncontrolled expansion of livestock into natural areas, especially in poor communities where livestock operations are small but have a high impact on their economy [13]. These pastures, often further from households, are less guarded, and fencing or herding livestock during vulnerable times may be too costly for owners, making them more susceptible to predation events [65]. Under this assumption, human–wildlife conflicts are likely to increase, unless minimizing livestock losses is a top priority. The implementation of measures to reduce or avoid livestock losses should generate support from the local population for the conservation of large carnivores proportional to the effectiveness of the mitigation [60]. A recent review of studies on the management tools used to reduce carnivores–livestock conflicts suggested that the effectiveness of lethal methods to protect livestock (killing predators) often fail and have not been tested with sufficient scientific rigor, and, in addition to legal considerations, have undesirable consequences for ecosystem stability and ethical controversies. In contrast, non-lethal methods associated with the presence of livestock guard dogs (LGDs), visual deterrents (fladry), fences and night corrals, or herdsmen demonstrated preventive effects on livestock predation [66,67]. Paradoxically, the use of specialist LGDs could benefit both farmers and wildlife by reducing livestock predation and thus retaliatory poaching [68]. To achieve these objectives, it is necessary to provide guidelines for the training of naïve puppies to LGDs, implement health and population control programs for LGDs, and prevent LGDs from roaming freely beyond farm boundaries [69,70]. In cases where an individual exhibits non-defensive behavior towards non-target species (e.g., attacks on wild herbivores), it must be removed from the herd to minimize harm to wildlife [71]. Additionally, the circulation of dogs between wild environments and rural areas facilitates the transmission of pathogens [72]. In Ecuador, dogs can carry a wide variety of pathogenic organisms, including viruses [73], bacteria [74], and parasites [75] of zoonotic concern. It is therefore essential that dog populations are controlled through responsible dog ownership in order to balance the issues and benefits that arise from the human–dog relationship.

A major challenge in carnivore conservation worldwide is identifying priority human–carnivore conflict sites where mitigation efforts would be most effective. Livestock predation often recurs in areas where carnivores attacked in the past, so spatially mapping these events can be useful for predicting a livestock predation risk hotspot [76]. However, a comprehensive long-term strategy requires not only implementing technical measures that identify and reduce attacks on livestock but also the involvement of local stakeholders. Interviews conducted by [60,65] within the predation conflict zone to examine people’s perceptions of Andean bears and jaguars revealed that young people with little formal education, who are exposed to other people’s experiences or anecdotal stories, are most likely to have negative attitudes towards large carnivores. False information with limited education sources to refute these perceptions makes them more susceptible to exaggerated misperceptions, suggesting that this group could potentially benefit from involvement in environmental education programs. Additionally, local communities are sometimes unaware of the threat dogs pose to their livestock, so it is important to raise public awareness of this issue [10]. Sometimes, dog predation is not only responsible for known livestock losses but actual levels of dog predation are also likely to be higher than the levels estimated from surveys [9]. In this sense, since this is the first study that evaluates the role of dogs on livestock attacks, it is necessary for policymakers to implement forensic DNA analysis in livestock predation events as legislative measure to assess how common the problem is in the region.

## 5. Conclusions

As human-dominated landscapes expand in Ecuador, the need to find innovative and effective solutions to mitigate the negative impacts of human–wildlife interactions is a conservation priority [60]. The Ecuadorian government is making efforts to manage livestock attacks [52], but the lack of infrastructure, technology, and financial resources is one of the greatest challenges that Ecuador must face in the coming years to reduce biodiversity loss [77]. Our research develops for the first time a non-invasive, easy-to-use tool for the reliable identification of apex predators in the Andean region and shows the involvement of domestic dogs in four of the six attacks (66.7%) analyzed in this study, which likely exacerbates the human–carnivore conflict. In addition, we recommend the implementation of measures to mitigate livestock predation, including the presence of guard-dogs, visual deterrents, fences, and herdsmen, as well as the control of free-ranging dogs. Spatially mapping these events can also be useful to predict livestock predation risk hotspots and establish specific management measures for each predator species. However, although this research provides baseline information that could be useful for livestock–carnivore coexistence efforts in Ecuador and neighboring countries, there are some limitations of this study that must be considered. In the future, it is necessary to implement measures for the timely identification of livestock attacks that allow for increasing the number of samples analyzed and reducing the time between a predation event and sample collection to improve the detection rate.

## Figures and Tables

**Table 1:** Standardized protocols for amplification of species-specific DNA regions for Andean bear, jaguar, puma, and domestic dog.

Species	DNA Region	Reference Literature	Standardized Protocol
Andean bear (*Tremarctos ornatus*)	COI	[54]	A 25 μL reaction system containing 1X PCR buffer, 1.5 mM MgCl_2_, 0.2 mM dNTPs, 0.2 mM of each primer, and 1.5 U Platinum^®^ Taq DNA polymerase. The amplification protocol was an initial denaturation step at 94 °C for 5 min, followed by 35 cycles of 94 °C denaturation for 45 s, 58 °C annealing for 1 min, 72 °C extension for 1 min, with a final extension step at 72 °C for 10 min.
Jaguar (*Panthera onca*)	12S	[55]	A 15 μL reaction system containing 1X PCR buffer, 1 mM MgCl_2_, 0.2 mM dNTPs, 0.5 μM of each primer, and 1U of Platinum^®^ Taq DNA polymerase. The amplification protocol was an initial denaturation step at 95 °C for 10 min, followed by 35 cycles of 95 °C denaturation for 30 s, 54 °C annealing for 30 s, 72 °C extension for 45 s, with a final extension step at 72 °C for 10 min.
Puma (*Puma concolor*)	16S	[56]	A 15 μL reaction system containing 1X PCR buffer, 2 mM MgCl_2_, 0.2 mM dNTPs, 0.5 μM of each primer, and 1U of Platinum^®^ Taq DNA polymerase. The amplification protocol was an initial denaturation step at 95 °C for 3 min, followed by 40 cycles of 94 °C denaturation for 30 s, 53 °C annealing for 45 s, 72 °C extension for 1 min, with a final extension step at 72 °C for 5 min.
Domestic dog (*Canis familiaris*)	CytB	[57]	A 15 μL reaction system containing 1X PCR buffer, 1.5 mM MgCl_2_, 0.2 mM dNTPs, 0.5 μM of each primer, and 1.5 U of Platinum^®^ Taq DNA polymerase. The amplification protocol was an initial denaturation step at 95 °C for 5 min, followed by 35 cycles of 94 °C denaturation for 30 s, 60 °C annealing for 30 s, 72 °C extension for 30 s, with a final extension step at 72 °C for 10 min.

**Table 2:** Results of controlled assays to test the standardized protocols. Five blind assays were conducted using samples collected from food chewed by individuals of each species (captive Andean bears, jaguars, and pumas, as well as domestic dogs). Each X marks the species that was identified using the standardized DNA extraction and PCR protocols.

	Species Identification			
Blind Assay Number	Andean Bear	Jaguar	Puma	Domestic Dog
1	X		X	X
2	X			X
3	X	X	X	X
4				X
5	X			X

**Table 3:** Detection of DNA from livestock attacks in four different events. Samples were taken from livestock wounds, DNA was extracted from them, and standardized PCR protocols were used.

Attack #	Site of the Attack	Approximate Date of Attack	Date of Sample Collection	Animal and Part of the Body from Which Sample Was Taken	Detected Animal Using Species-Specific DNA Markers
1	Nono, Pichincha	No information	27 December 2021	Cow—neck	Domestic dog, puma
				Cow—inferior extremity	None
2	Yanahurco grande, Cotopaxi	20 February 2022 (20:00)	21 February 2022	Sheep 1—unknown	Domestic dog
				Sheep 2—unknown	Domestic dog
				Sheep 3—unknown	Domestic dog
				Sheep 4—unknown	Domestic dog
3	Paluguillo, Pichincha	On or before 25 December 2022	27 December 2022	Sheep-Face	None
4	Nono bajo, Pichincha	On or before 23 April 2023	26 April 2023	Cow—face	None
				Cow—thorax	None
				Cow—leg	None
5	Chilla, El Oro	22 December 2023	22 December 2023	Sheep—neck	Domestic dog
6	Las Villegas, Santo Domingo	14 January 2024	14 January 2024	Mule—mouth, nose, head, hip (individual sample origin was not identified)	Domestic dog

## Data Availability

Data are contained within the article.
